# Swelling of Hydrogels Based on Carboxymethylated Starch and Poly(Acrylic Acid): Nonlinear Rheological Approach

**DOI:** 10.3390/polym12112564

**Published:** 2020-10-31

**Authors:** Grzegorz Kowalski, Paweł Ptaszek, Łukasz Kuterasiński

**Affiliations:** 1 Faculty of Food Technology, Department of Engineering and Machinery for Food Industry, University of Agriculture in Kraków, 30-149 Kraków, Poland; p.ptaszek@urk.edu.pl; 2 Jerzy Haber Institute of Catalysis and Surface Chemistry, Polish Academy of Sciences, 30-239 Kraków, Poland; nckutera@cyf-kr.edu.pl

**Keywords:** hydrogels, acrylic acid, starch, LAOS, nonlinear rheology

## Abstract

In this paper, the authors discuss the results of research on the preparation and properties of superabsorbent hydrogels based on carboxymethylated high-amylose corn starches. They were obtained by graft copolymerisation (in an aqueous environment) of acrylic acid and its sodium salt onto modified starches (with various substitution degrees DS = 0.2 and 0.8), using potassium persulfate as an initiator and *N*,*N*’-methylenebisacrylamide (MBA) as a cross-linker. Modified starches, with various DS, were used to synthesise two series of hydrogels with varying molar ratios of cross-linkers to monomers. The swelling behaviour of hydrogels was studied and their properties were estimated using the kinetic equation. The occurrence of starch–polyacrylic acid covalent interactions was demonstrated by FTIR analysis. Nonlinear rheological methods have proved to be very effective in assessing the mechanical properties of hydrogels. LAOS (large -amplitude oscillatory shear) analysis allowed the determination of the durability of the gel structure as a function of the amount of absorbed water.

## 1. Introduction

Hydrogels obtained on the basis of acrylic acid are widely known and used as superabsorbents and are characterised by the fact that they are able to absorb (store) significant amounts of water. The most efficient hydrogels can absorb up to 1000 times more water than their weight [1]. These materials are obtained mainly by radical polymerisation of acrylic monomers, e.g., acrylic acid [2], acrylic acid salts [3], acrylamide [4] or methyl methacrylate. This reaction occurs in the presence of a small number of cross-linkers, the amount of which in the process, allows the control of the properties of the final product.

Starch is a renewable and biodegradable system consisting of two biopolymers: Linear amylose and a branched amylopectin [5,6]. The ratio of amylose to amylopectin depends on the botanical origin of the starch [5,6,7,8,9,10,11,12]. This polysaccharide is an essential component of the human diet, but may also find a variety of nonfood uses [13,14]. One of them may be the implementation of starch as a base onto which polyacrylic acid is grafted [15,16,17,18,19,20,21]. High-amylose corn starch is interesting in terms of its use for the synthesis of hydrogels. The predominance of linear chains in the structure of starch grains results in better associations of the chains, and thus, the formation of a much larger number of hydrogen bonds, resulting in greater structure ordering compared to native starches [5,6,22], e.g., waxy (with higher amylopectin content). In order to increase solubility in water, the starch undergoes various modifications, including the process of carboxymethylation [23,24,25]. Such a modification should also improve the water-binding capacity of the hydrogel.

Both starch and polyacrylic acid contain a large number of hydrophilic groups in the form of hydroxyl and carboxyl groups. This property makes such polymers a good base for the synthesis of new hydrogels with increased absorption properties.

Polyacrylic acid copolymers and starch demonstrate different properties in the gel polymer network depending on composition (mutual proportions). This is because there are two types of bonds in the system: Typically covalent (chemical) and weaker hydrogen bonds [6,8,12]. The presence of two types of bonds primarily affects the mechanical properties of the resulting gels. For this reason, it is very important to know the rheological properties of these types of systems with varying degrees of water absorption. Knowledge of these properties can facilitate the description of the entire hydrogel-water structure strength as a function of the amount of bound water [26,27].

Currently, a very popular research technique in the analysis of soft matter properties is the use of large-amplitude oscillatory shear (LAOS) [28]. This technique allows the tracking of changes in material structure regarding nonlinear deformation [28]. Based on LAOS methods, very broad analysis of material behaviour under the influence of high mechanical deformation values can be conducted. Acquisition of information may be performed on the basis of Fourier spectrum analysis or analysis of Lissajous figures [29,30]. Based on these data, it is possible to describe the degree of nonlinearity of the material’s response as a function of deformation amplitude, the amount of mechanical energy dissipated, and to assess parameters describing purely elastic and purely viscous behaviour in relation to the area of nonlinear viscoelasticity.

Parameters obtained in this manner have a direct impact on the actual behaviour of the material in conditions of the production process or during its use (exploitation) [30,31].

The aim of the work was to produce hydrogels based on carboxymethylated starch and acrylic acid, to determine their basic physicochemical parameters and to examine the nonlinear rheological properties of the produced hydrogels using LAOS methods with varying degrees of water absorption.

Based on our knowledge, there were few studies on the preparation of these types of hydrogels [27,32], but no attempt was made to determine the correlation between the degree of starch substitution, the degree of cross-linking, water content, and their mechanical properties.

## 2. Materials and Methods

### 2.1. Materials

In this work, Hylon VII (National Starch, Westchester, IL, USA) high-amylose corn starch was used for carboxymethylation. The amylose content of the starch was 70%. Sodium monochloroacetate (SMCA), acrylic acid and methylenebisacrylamide (MBA) were purchased from Fluka (Buchs, Switzerland). Potassium persulfate, methanol, isopropanol and NaOH were purchased from POCH (Gliwice, Poland). All reagents were of analytical grade.

### 2.2. Preparation of Carboxymethyl Starch

Carboxymethylation of corn starch was carried out in a 1 L three-necked round-bottom flask, equipped with a motor-driven stirrer [33]. Sodium hydroxide (25 g) was added to the flask containing distilled water (104 mL), and the mixture was stirred at 250 rpm until NaOH was dissolved. Isopropanol (650 mL) was added to the solution and the temperature was then raised to 40 °C; next, 70 g (dry weight) of starch was added and stirred at 400 rpm. The reaction mixture was purged with nitrogen in order to minimise side reactions. After 1 h of stirring, 63 g of SMCA was added to the mixture and the reaction was carried out by stirring in a nitrogen atmosphere for 3 h. After completion of the reaction, the starch slurry was filtered, suspended in methanol and neutralised with acetic acid. The precipitate was washed several times with 85% methanol-water solution until the silver nitrate test for chloride presence in filtrate was negative. The precipitate was suspended in acetone, stirred for 20 min, filtered and dried in an oven at 40 °C for 48 h.

### 2.3. Hydrogel Synthesis

Aqueous solutions of the acrylic acid monomer (30 g in 7.6 g of water) were partially neutralised (30% of the carboxyl groups) with 5.008 g of sodium hydroxide dissolved in 14.5 g of water. Then, solutions of potassium persulfate (0.12 g in 3.2 g of water) and *N*,*N*’-methylenebisacrylamide (0.0047, 0.0095, 0.0280 and 0.0480 mol % with respect to monomer) dissolved in 3.2 g of water were prepared. The combined solutions, excluding the initiator, were poured into a 2 L three-necked flask immersed in a water bath and heated to 80 °C. The solution was flushed with nitrogen. After that, 8 g of carboxymethylated starch was added and the mixture was stirred with a magnetic stirrer (400 rpm). Finally, the initiator solution was added and the reaction mixture was left to stir until the viscosity of the solution was too high to continue further mixing. After 1 h when the reaction was completed, the mixture was cooled down to room temperature. The resulting rubbery gel was removed from the flask and was cut into small irregular pieces (5–10 mm) with a knife and scissors. Then, the pieces were dehydrated in methanol, and after its removal, the solution samples were transferred to Petri dishes and heated in an air-circulating oven at 60 °C for a minimum of 48 h until reaching dryness. The dried gels were ground using a mini-grinder (laboratory scale) and later screened. The classified particles were stored at ambient temperature in tightly stoppered plastic containers and used in further experiments.

### 2.4. Determination of Substitution Degree

Substitution degree (DS) of starch with carboxymethyl groups was determined via the back-titration method. The excess of NaOH was titrated with hydrochloric acid as described in the procedures given below.

Approximately 3 g of carboxymethylated starch sodium salt (Na–CMS) was dispersed in 100 mL of acetone; then, 50 mL of 1 M aqueous HCl was added. The dispersion was stirred for 30 min to completely convert the sodium salt form of carboxymethyl starch to its protonated form (H–CMS). The suspension was filtered and washed with 85% (*v/v*) methanol to remove excess HCl until the pH of the solution was neutral. Afterwards, the precipitate was dispersed in acetone and stirred for 20 min. Then, the dispersion was filtered and the acidic form of CMS was dried at 40 °C for 24 h. To determine the DS, 20 mL of 0.2 M NaOH and 50 mL of distilled water were added to 0.5 g of the H-CMS sample. The mixture was stirred overnight. Following that, the solution was transferred to a 100 mL volumetric flask, which was filled up to the 100 mL mark with distilled water. Then, 25 mL of the solution was diluted with 100 mL of distilled water in a 250 mL Erlenmayer flask. The excess NaOH was back-titrated with 0.1 M of HCl solution in the presence of phenolphthalein. The analysis was repeated three times, and the average DS value calculated. The degree of substitution was calculated from Equations (1) and (2):

(1) $$\mathrm{DS}=\frac{M_{0}\cdot n_{\mathrm{COOH}}}{m_{\mathrm{ds}}-M_{r}n_{\mathrm{COOH}}},$$

(2) $$n_{\mathrm{COOH}}=\left(V_{b}-V\right)\times c_{\mathrm{HCl}}\times 4,$$

where:

*M*_0_—molar mass of anhydroglucose unit (162 g/mol),
*M*_r_—molar mass of carboxymethyl residue (58 g/mol),
*m*_ds_—weight of modified starch (g),
*n*_COOH_—amount of HCl used in titration (mol),
*V*_b_—volume of HCl used for titration of the blank (L),
*V*—volume of HCl used for titration of the sample (L),
*c*_HCl_—concentration of HCl solution (mol/L).

### 2.5. Swelling Properties

A sample of dried hydrogel (0.3 g) was immersed in 200 mL of distilled water (200 mL) at room temperature for a defined period of time. During this time, the samples were stirred with a magnetic stirrer (300 rpm). To speed up the filtration process, the dispersion of water-swollen gel particles was filtered through a Büchner funnel and filtered samples were weighed. For absorption measuring rate, the samples were taken from the solution after a precisely defined period of time. For each sample, measurements were repeated three times. The swelling ratio was calculated using Equation (3):

(3) $$W_{t}=\frac{M_{w}-M_{d}}{M_{d}},$$

where:

*W*_t_—swelling ratio at time *t* (g·H_2_O/g·dry gel),
*M*_w_—weight of swollen hydrogel at time *t* (g),
*M*_p_—weight of dried gel (g).

Changes in swelling properties were estimated with the following kinetic equation: [27]

(4) $$W\left(t\right)=W_{\infty }\frac{k_{1}\cdot t}{{\left(1+K\cdot t\right)}^{a}}=W_{\max}\times e^{-bx}\frac{k_{1}\cdot t}{{\left(1+K\cdot t\right)}^{a}},$$

where:

*x*—molar ratio of cross-linker to monomer,
*k_1_*—water swelling rate constant by hydrogel (g/(g·min)),
$W_{\infty }-$ water absorption at equilibrium,
*K* (min^−1^) and *a*—determine denominator value representing resistance of diffusion process.

Estimation of Equation (4) parameters was carried out using the Marquardt–Levenberg method.

### 2.6. Sol Content Measurement

Into a 1 L beaker containing 100 mL of 1.0% sodium chloride solution, 0.1 g (±0.0001 g) of dried superabsorbent was poured. The hydrogel dispersion was left on a shaker for 72 h so that the soluble fraction would transfer to the solution. After this time, it was left until the solution became clear. The supernatant from the precipitate was filtered and UV/VIS analysis was carried out within a wavelength range of 180–300 nm with a single-beam spectrophotometer (Labomed, Inc. UV-VIS 2800, Los Angeles, CA, USA). The measurements were performed in a quartz cuvette with a light path length of 1 cm. A 1% aqueous NaCl solution was used as a reference. The apparatus was calibrated on the basis of acrylic acid solutions with given concentrations. In the tested samples, maximal absorption was observed at a wavelength of approximately 208 nm.

### 2.7. FT-IR Spectroscopy

The FT-IR experiments were carried out using the Nicolet 6700 spectrometer with an MCT (mercury cadmium telluride) detector and ATR adapter from Thermo Scientific (Madison, WI, USA). The scanning range of the IR spectra was 650–4000 cm^−1^ and 32 scans were obtained for each spectrum.

### 2.8. Rheological Measurements

Measurements were performed using the RS6000 rheometer (Haake, Vreden, Germany). The set of sensors and the size of the measurement gap were empirically selected in the course of the tests. Best results were acquired for the plate-plate-type sensor. 

Rheological measurements were conducted on samples subjected to a previously controlled swelling process in an aquatic environment. A strictly defined amount of water was added to each of the tested samples. The amount of water used for swelling was determined in such a manner that the amount of water absorbed by individual samples was always identical, i.e., 5, 10, 25, 50 and 100 g of water per 1 g of the hydrogel sample. For all tested samples, the amount of water added did not exceed maximal water absorption for a given hydrogel.

All rheological measurements were carried out using an RS6000 rheometer (Haake, Vreden, Germany) equipped with a plate-plate geometry system (diameter of plate din = 35 mm) at a f = 1 Hz frequency and for amplitudes within the γ_o_ range of 0.0005–50. The rheological tests were preceded by multiple trials to eliminate the slip of the investigated material on the plate-plate walls. The preliminary studies included tests with the following measurement units: Corrugated plate-plate type, plate-plate with attached high-gradation sandpaper (3000) and simple plate-plate. The next step in the research was to select a measurement gap, as this particular parameter plays a key role in tests on dispersed systems. A set of measurement units and the size of the measurement gap were empirically determined in the course of the tests. The accepted reproducibility of the results was at the level of 95%. This led to the elimination of both the corrugated and flat plate-plate devices. Best results were obtained for the simple plate-plate. As the plate-plate-type unit was found easier in operation, it was decided to apply this option, combined with the measurement gap of 2 mm.

The appropriate rheological measurements were conducted in triplicate at 25 °C. All the time series obtained during the measurements were composed of 15 periods, of which the last 6 periods were analysed.

Simultaneously, measurements of the signal-to-noise ratio (S/N) were performed. This is defined as the ratio of the amplitude of the highest peak, divided by the standard deviation of the noise [31]. Preliminary rheological tests were carried out using the described plate-plate geometry. Calculation of the signal-to-noise ratio (S/N) was done as per procedures proposed by Wilhelm [31,34]. As the first step, rheological testing was performed for a Newtonian liquid (glycerine) in order to determine the intensity of nonlinear effects generated by the rheometer. Based on the resulting Fourier spectrum, the ratio of S/N was estimated at 9 × 10^4^. The same procedure was performed on the studied systems and, in this case, the S/N ratio was estimated at 8.5 × 10^4^.

The measurement concept based on the LAOS technique is analogous to the measurements which employ the application of small amplitudes within the range of linear viscoelasticity. This concept consists of subjecting the investigated material to the time-variable strain. Due to the ease of its analysis, it is most commonly a sinusoidal signal which adopts the following form [28]:

(5) $$\gamma \left(\omega t\right)=\gamma_{0}sin\left(\omega t\right),$$

For shear stress, the response can be expressed with the help of the following harmonic function:

(6) $$\tau \left(t;\omega ,\gamma_{0}\right)=\gamma_{0}\sum_{nodd}\left[G_{n}^{’}\left(n\omega t\right)+G_{n}^{’’}\left(\omega ,\gamma_{0}\right)\cdot cos\left(n\omega t\right)\right],$$

In the case of small strains, there is only one harmonic present, and hence, *G’_n_* and *G”_n_* become real (*G’*) and imaginary (*G”*) parts of the complex elastic modulus (G* = G’+jG”), well-known from research on linear viscoelasticity. As high values of deformation amplitudes are applied, a larger number of harmonics are observed which are typical for the nonlinear response of the material. 

Analysis of the results was divided into two parts. The first comprised analysis of G’ and G” moduli dependencies as the function of amplitude. The second part directly referred to the analysis of the time series. 

The method of 2-D Lissajous figure geometrical decomposition, proposed by Cho et al., was applied in this study [30]. According to the premise of this method, stresses (τ) can be subjected to decomposition, as expressed by the following equation:

(7) $$\begin{array}{c} \tau \left(x,y\right)=\frac{\tau \left(x,y\right)-\tau \left(-x,y\right)}{2}+\frac{\tau \left(x,y\right)-\tau \left(x,-y\right)}{2}=\tau^{\prime }\left(x\right)+\tau^{\prime \prime }\left(y\right),\;\\ x=\gamma ,y=\gamma /\omega ; \end{array}$$

where:

τ’ (*x*;γ*_0_*,ω)—elastic stress value,
τ’’(*y;*γ*_0_*,ω)—viscous stress value.

The curves split the Lissajous figure into two parts of equal area. The advantage of this approach is the nonlinear signal decomposition (obtained experimentally) into parts corresponding to the elastic and viscous properties without the need to apply any constitutive equations [30].

The curves may be subjected to further decomposition. For this purpose, two methods are used: The first applies regression analysis and the least squares method [30], whereas the second procedure is based on Chebyshev polynomials of the first kind [29], obtained according to the recurrence rule:

(8) $$\begin{array}{c} T_{0}\left(x\right)=1\\ T_{1}\left(x\right)=x\\ T_{n}\left(x\right)=2x\cdot T_{n-1}\left(x\right)-T_{n-2}\left(x\right) \end{array},$$

Then, τ’ and τ” may be expressed by the following dependencies:

(9) $$\begin{array}{c} \tau^{\prime }\left(\bar{x}\right)=\gamma_{0}\underset{n:odd}{\sum }e_{n}\left(\omega ,\gamma_{0}\right)\cdot T_{n}\left(\bar{x}\right)\\ \tau^{\prime \prime }\left(\bar{y}\right)=\dot{\gamma_{0}}\underset{n:odd}{\sum }\nu_{n}\left(\omega ,\gamma_{0}\right)\cdot T_{n}\left(\bar{y}\right) \end{array}$$

where:

$$\bar{x}=x/\gamma_{0}=\gamma /\gamma_{0},\bar{y}=y/\gamma_{0}=\dot{\gamma }/{\dot{\gamma }}_{0}.$$

Scaling is a result of orthogonality conditions according to Chebyshev polynomials [35]. The coefficients *e*_n_ and *v*_n_ are called Chebyshev weighted coefficients and they correspond to elastic and viscous parts in nonlinear viscoelasticity, respectively. It should be noted that Fourier coefficients (G’_n_, G”_n_) in Equation (6) fully characterise the response of the material within the time range; however, the physical interpretation of the higher harmonics may only be carried out based on the *e_n_* and *v_n_* Chebyshev coefficients [28,29].

Chebyshev coefficients may have both positive and negative values [28]. Usually, the interpretation of the liquid’s properties can be carried out by determining the values of *e_3_* and *v_3_*:

$$e_{3}=\{\begin{array}{cc} 0 & \mathrm{strain}-\mathrm{stiffening}\\ 0 & \mathrm{linearelastic}\\ 0 & \mathrm{strain}-\mathrm{softening} \end{array}v_{3}=\{\begin{array}{cc} 0 & \mathrm{shear}-\mathrm{thickening}\\ 0 & \mathrm{linearviscous}\;\left(\mathrm{Newtonian}\right)\\ 0 & \mathrm{shear}-\mathrm{thinning} \end{array}$$

## 3. Results and Discussion

As part of the research described in this article, hybrid hydrogels having very high water absorption were synthesised. Superabsorbents based on modified carboxymethyl, high-amylose maize starch and partially neutralised acrylic acid were obtained via radical copolymerisation of the monomer, modified starch and *N*,*N*’-methylenebisacrylamide cross-linking agent (Table 1). The modification of starch by introducing additional groups is intended to change its properties. In the described research, high-amylose (about 70%) corn starch was etherified with sodium monochloroacetate in an alkaline medium. Carboxymethylated starches with two different degrees of substitution were obtained as a result of the reactions carried out under the synthesis reaction conditions described by Lawal [33]. The determined degrees of substitution in starch, by reverse titration, were 0.2 (samples designated CMS1SK1-CMS1SK4) and 0.8 (samples designated CMS2SK1-CMS2SK4), respectively. As a result of the etherification reaction, some hydroxyl groups were substituted using carboxymethyl groups present in the form of anions. This type of modifying reaction increases the affinity of the modified polysaccharide for water, and therefore, higher water absorption of hydrogels should be expected for these polymers. In this article, the impact of two important parameters on the water absorption and rheological properties of superabsorbents, i.e., the degree of substitution of carboxymethyl starch, as well as the concentration of the cross-linking agent, is presented. The amount of cross-linking agent was based on our previous research [27,36].

In order to test water absorption, hydrogel samples were placed in distilled water for a strictly defined period, i.e., 5, 10, 20, 40, 60 and 120 min. After this time, nonabsorbed water was removed from the samples by filtration. 

**Figure 1:**
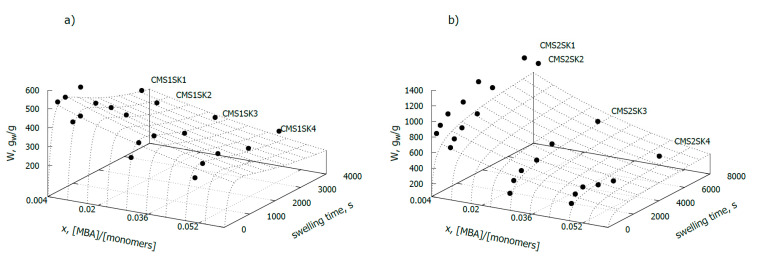
Swelling capacity as a function of cross-linker-to-monomer ratio and swelling time, for hydrogels based on modified starch: (**a**) Substitution degree (DS) = 0.2, (**b**) DS = 0.8.

Samples of hydrogels with a low degree of carboxymethyl substitution (Figure 1a) were characterised by much lower water absorption than the samples for which the degree of substitution was 0.8 (Figure 1b). In the case of samples with a substitution level of 0.2 (CMS1SK1-CMS1SK4), the water absorption at steady state varies from 257 to 346 g/g, depending on the amount of initiator added. For samples in which the degree of substitution with carboxymethyl groups is 0.8 and with a similar amount of cross-linking agent used for the reaction, the measured water absorption in equilibrium varies between 232 and 1203 g/g. The amount of absorbed water strongly depends on the amount of cross-linker used in the reaction, and as expected, it experiences a decrease with increasing amounts. This relationship was observed for both series of tested hydrogels. Hydrogels obtained in the presence of a greater amount of cross-linking agent have a lower water absorption rate. This phenomenon is easily explained because the diffusion of water deep into the hydrogel network with high cross-linking density is difficult. This phenomenon is related to the fact that the structure of the hydrogel with high cross-linking density is more compact, and the “eyelets” in the network are much smaller in size.

It should also be noted that cross-linking density has a much greater effect on the hydrogel’s ability to absorb water, in the case of samples for which DS = 0.8 (CMS2 series) (Figure 1). The value of parameter b (Equation (4)), related to the amount of cross-linker used in hydrogel synthesis, for samples with DS = 0.8, is nearly three times higher than for samples with a very low degree of substitution (DS = 0.2) and is 33.8 and 12.5, respectively (Table 2).

**Figure 2:**
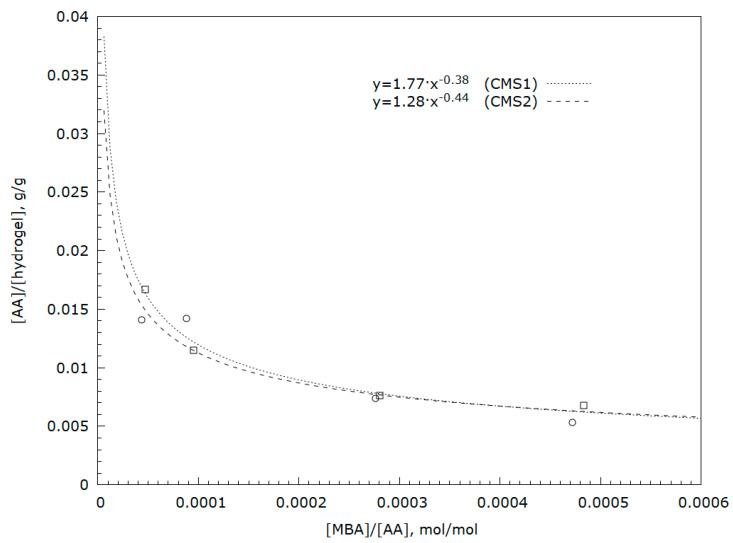
Dependence of nonreacted acrylic acid amount on cross-linker concentration in hydrogel samples with a degree of substitution: (**a**) Dotted line DS = 0.2 CMS1 and (**b**) dashed line DS = 0.8 CMS2.

Analysis of soluble hydrogel fractions using UV-VIS spectroscopy (analysis of the characteristic band for carbon–carbon double bonds) showed the presence of small amounts of unreacted acrylic acid or soluble short-chain acrylic acid oligomers (Figure 2). As the starch gel molecules are immobile, the possibility of reaction with acrylic acid depends on the distance of the molecules of this acid from the hydroxyl groups of starch. Increasing the amount of monomer in the network results in greater hydrophilicity of the resulting polymer which, in turn, allows a product to be obtained with greater affinity for water [4]. A greater amount of unreacted acid means a lower degree of monomer conversion and may result in a lower degree of substitution in the starch matrix. Based on the experiment, it may be concluded that large amounts of cross-linking agents have a positive effect on the degree of acrylic acid conversion. This is confirmed in the research by Lawal et al. [33]. It has also been shown that this relationship has power characteristics [37] (Figure 2).

**Figure 3:**
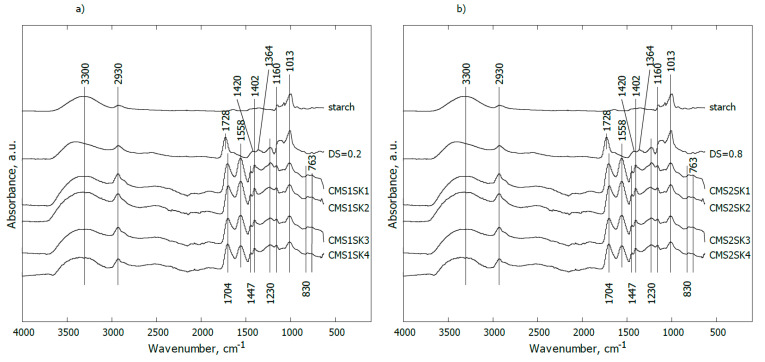
FTIR spectra of starch and hydrogels based on carboxymethylated high-amylose corn starch: (**a**) CMS1SK1-CMS1SK4, (**b**) CMS2SK1-CMS2SK4.

In Figure 3a, the IR spectra of pure starch (line starch), modified starch containing carboxymethyl groups in the anhydroglucose unit with DS = 0.2, hydrogel based on modified starch, cross-linker and AA (acrylic acid) polymer with different chemical compositions (lines CMS1SK1-CMS1SK4) are shown. The content of starch and the AA polymer was constant while the amount of cross-linker was gradually changed (from 0.0047 to 0.0483 mol %). 

For pure starch, the band at 3300 cm^−1^ can be assigned to the OH stretching mode, while the signal observed at 2930 cm^−1^ may be attributed to stretching vibrations of C–H groups. The occurrence of distinct bands observed together in the range of 1160–900 cm^−1^ is associated with C–O–C stretching vibrations or with –CH–OH in aliphatic cyclic secondary alcohol.

Substitution of the hydroxyl groups in the anhydroglucose unit (AGU) with the carboxymethyl groups (line CMS1SK1) led to the appearance of a band at 1728 cm^−1^ corresponding to asymmetric C=O stretching vibrations present in carboxylic ions (COO^−^). The weak shoulder at ca. 1640 cm^−1^ (not indicated) may be assigned to COO^−^ symmetrical stretching vibrations or is implied from water bending vibrations. The small band at ca. 1420 cm^−1^ probably comes from carboxyl asymmetrical stretching vibrations. In turn, the signal at 1364 cm^−1^ may be attributed to bending vibrations of C–H or O–H groups in the AGU. A distinct band at 1230 cm^−1^ may be interpreted as a result of C–O–C stretching vibrations between starch and carboxymethyl groups. 

In the case of the mixture containing a minimal content of cross-linker (line CMS1SK3), the broad band at ca. 3330 cm^−1^ can be assigned to the OH stretching mode (from acrylic acid, starch, water), and overlaps with the band coming from N–H stretching vibrations (from the cross-linker). The signal observed at ca. 2930 cm^−1^ could be attributed to stretching vibrations of C–H groups. The bands at ca. 1704 and 1447 cm^−1^ may be assigned to COO^−^ symmetrical and asymmetrical stretching vibrations, respectively. Carboxylic ions occur either in an AA polymer or in the AGU. The occurrence of the band at 1447 cm^−1^ may also reflect scissoring –CH_2_– vibrations. The maximal value found at 1558 cm^−1^ comes from amide II. The presence of bands at 1402 and 1162 cm^−1^ is typical for –CH_2_– and/or OH bending, and stretching vibrations of C=C in (C=C)–(C=O) in cross-linkers, respectively [38,39,40,41,42,43,44,45]. The maxima found at ca. 1230 and 1013 cm^−1^ are probably associated with C–O–C stretching vibrations or with –CH–OH/CH_2_–OH in the AGU. C–O–C groupings are also formed in a reaction between starch and AA polymers in the presence of a cross-linker and/or may be generated through the substitution of hydroxyls by the carboxymethyl groups in the AGU. 

Another effect of the reaction between modified starch (containing carboxymethyl groups in the AGU), the AA polymer and cross-linker may be disappearance of the band at 1420 and 1364 cm^−1^ coming from carboxyl asymmetrical stretching vibrations and bending vibrations of C–H or O–H groups in the AGU, respectively. 

The presence of the maximal 830 cm^−1^ value is connected with bending vibrations of carboxylic ions, while the band at 763 cm^−1^ may be associated with rocking vibrations of –CH_2_– groups [38,39,40,41,42,43,44,45].

Increasing the amount of cross-linker in relation to other components did not change the appearance of the IR spectra (lines CMS1SK2–CMS1SK4).

In Figure 3b, the IR spectra of the mixture of starch, cross-linker and AA polymer with an elevated degree of substitution of the hydroxyl groups in the anhydroglucose unit (AGU) with the carboxymethyl groups (DS = 0.8) are given. Neither increasing DS value nor higher cross-linker content results in noticeable changes for the presented IR spectra. 

### Rheology

**Figure 4:**
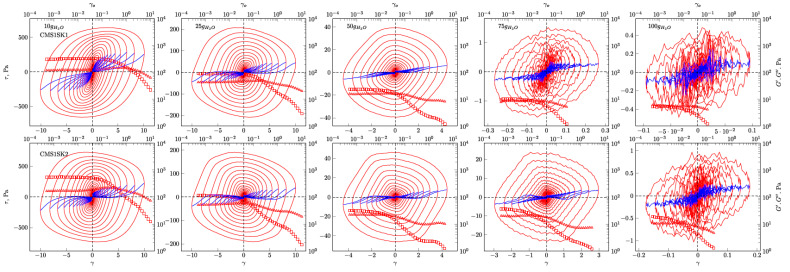
Elastic Lissajous figures and geometrical decomposition for CMS1SK1 and CMS1SK2 hydrogels. Red line—Lissajous figure, blue line—geometrical decomposition. □—*G’*, Δ—*G’’*.

**Figure 5:**
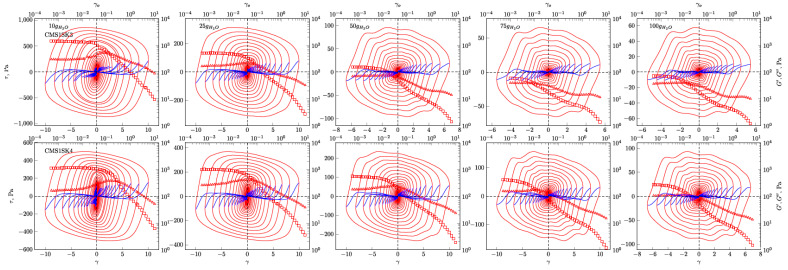
Elastic Lissajous figures and geometrical decomposition for CMS1SK3 and CMS1SK4 hydrogels. Red line—Lissajous figure, blue line—geometrical decomposition. □—*G’*, Δ—*G’’*.

**Figure 6:**
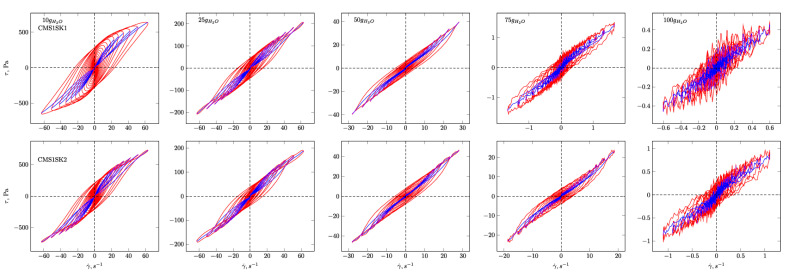
Viscous Lissajous figures and geometrical decomposition for CMS1SK1 and CMS1SK2 hydrogels. Red line—Lissajous figure, blue line—geometrical decomposition.

**Figure 7:**
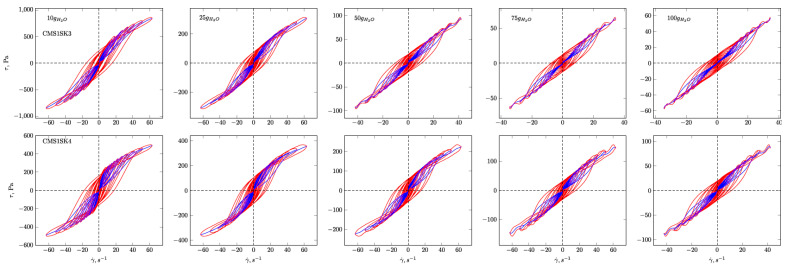
Viscous Lissajous figures and geometrical decomposition for CMS1SK3 and CMS1SK4 hydrogels. Red line—Lissajous figure, blue line—geometrical decomposition.

In Figure 4, Figure 5, Figure 6 and Figure 7, the G’ and G” dependencies as a function of strain amplitude and the elastic (Figure 4 and Figure 5) and viscous (Figure 6 and Figure 7) Lissajous figures corresponding to the description of elastic behaviour for the CMS1SK1 and CMS1K2, as well as CMS1SK3 and CMS1SK4 systems, are shown. In these figures, the rows represent the structure’s response to the amount of absorbed water, while the columns correspond to the appropriate amount of cross-linking agent. The G’ and G” dependencies as a function of strain amplitude demonstrate a typical relationship for cross-linked systems. As the amount of water bound in the structure increases, the range of linear viscoelasticity is significantly shortened until its complete disappearance. This phenomenon is more pronounced for CMS1SK1 and CMS1SK2, while the second system CMS1SK3 and CMS1SK4 is able to transfer higher mechanical loads. Furthermore, the module values (G’, G”) are reduced. This phenomenon results from stretching of the polymer network due to the presence of water. In systems containing the least amount of water, the network shows the smallest spontaneous deformation caused by the smallest tension in bonds. The increase in absorbed water content causes the entire system to swell and the individual chains forming the network to stretch. This is manifested by a reduction in energy storage capacity within the system, and thus, a shortening of the range of linear viscoelasticity and the value of the modules themselves (G’ and G”). Analysis of the Lissajous figures themselves confirms these phenomena. Initially, for small values of deformation amplitude, these figures have the shape of ellipses, which corresponds to typically viscoelastic behaviour. As the value of the deformation amplitude increases, the surface of the figure also experiences an increase, and the shape that is a regular figure (ellipse or rectangle) changes. This behaviour indicates a change in the system’s properties from elastic to tacky. This behaviour also confirms the course of G’ and G”, in which the intersection of these curves is observed. The values of G’ become smaller than G” (G’<G”). This means that more energy is dissipated than stored in one period. Further water absorption causes a clear reduction in the surface of Lissajous figures and a clear change in shape, similar to a rectangle. For large amounts of absorbed water, the measurement was only possible in a limited range of amplitudes, and the G’ and G” values were initially close to each other. In addition, the recorded measurement signal was characterised by the presence of disturbances that prevented further analysis. These phenomena result from the very large amount of water absorbed and the swelling of the polymer network. In these cases, the system’s ability to store mechanical energy is very limited, which is due to high tension in the polymer chains. Therefore, the area of linear viscoelasticity is small and the values of deformation amplitudes causing irreversible deformation and/or destruction of the tested material are small compared to previous cases. The analysis of Lissajous figures reveals one more phenomenon—an increase in the amount of cross-linking agent, and the content of bound water causes the Lissajous figure to rotate. Rotation is manifested by a change in the slope of the large axis regarding the figure relative to the axis of the coordinate system representing the deformation. For gels containing the smallest amounts of cross-linking agent, the maximal figure value is in the first quadrant of the coordinate system and the geometric decomposition line only changes sign once while passing through the centre of the coordinate system (the only exception is the CMS1SK1 and CMS1SK2 system containing 25 g of water, in which initially, along with the increase in amplitude, rotation of the whole figure follows). The increase in cross-linker content and the number of starch functional groups differentiates this behaviour. CMS1SK1 and CMS1SK2 show the maximum only in the first quarter of the coordinate system, the increase in water content in the structure causing the figure rotation described above. The CMS1SK3 and CMS1SK4 system exhibits completely different behaviour. The increase in water content causes overlapping of decomposition lines, which indicates a very similar shape of Lissajous figures. The system containing 75 g of water is characterised by identical shapes of figures, differing only in surface. A further increase in the water content of the structure causes rotations and decomposition lines to change sign several times. For systems containing a higher amount of cross-linking agent, the phenomena described above do not occur. The Lissajous figure maximum is in the second quadrant of the coordinate system, the lines of geometric decomposition changing sign repeatedly.

The analysis of Lissajous viscous figures also confirms the properties of the discussed systems described above. They are characterised by a typical spindle shape, and their surface becomes smaller as the viscous properties increase (deformation amplitude increases). Lissajous figures describing purely tacky behaviour also demonstrate the double-loop phenomenon with an increase in the amount of cross-linking agent and an increase in the content of absorbed water. This phenomenon undergoes evolution, and for some systems, several double-loops are visible. This shows a very complex response of the tested material to the applied deformation.

**Figure 8:**
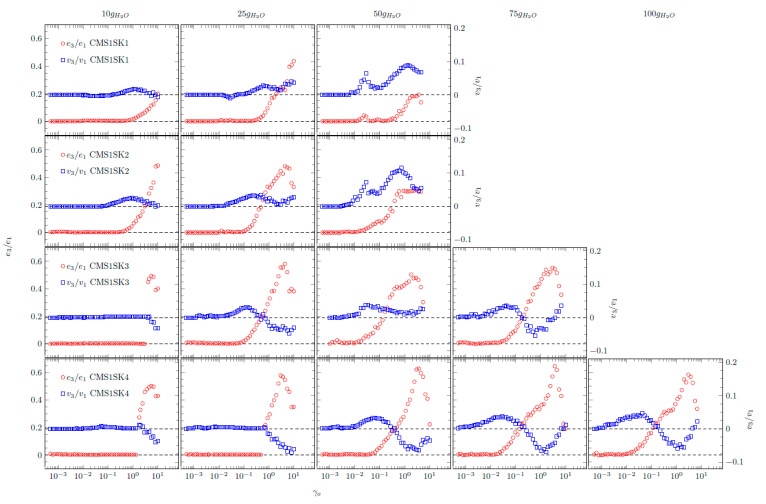
Chebyshev coefficients for investigated hydrogels.

Interesting behaviour is also shown by the relationships of normalised Chebyshev coefficients as a function of deformation amplitude (Figure 8). During the research, it was possible to obtain interpretable results for systems containing small and medium amounts of absorbed water. In the case of some samples (CMS1SK1, CMS1SK2 and CMS1SK3 for 75 and 100 g H_2_O), the calculated values of Chebyshev coefficients did not have a physical interpretation; therefore, they are not presented in Figure 8. The values of *e_3_* show values equal to or greater than zero for all the analysed systems, and some of them have characteristic maxima. This behaviour indicates that the tested systems exhibit strain stiffening. The *v_3_* coefficients assume values both greater and less than zero. This means that there is a change in the nature of these systems from compacted to diluted shear. Comparison of *e_3_* and *v_3_* waveforms shows that their behaviour is closely related. When an increase in the value of *e_3_* is observed, the maximal value appears on the *v_3_* curve. However, the maximum on curve *e_3_* corresponds to the minimum on curve *v_3_*.

Analysis regarding the influence of the amount of cross-linking agent on the nonlinear properties of the tested absorbents is also very interesting. An increase in the content of this factor causes the appearance of the characteristic maximum on the G” curve in the G’, G” intersection area as a function of strain amplitude. In addition, there is an increase in the value of modules G’ and G” themselves. This is due to the presence of more connections building the polymer network itself, allowing the accumulation of more mechanical energy within the structure. In addition, the amount of the factor does not significantly affect the magnitude of the linear viscoelastic range for a given content of absorbed water. The high content of cross-linking agent helps to improve the mechanical properties of the tested systems. In this case, there is no material damage caused by small values of deformation amplitude, and also, the value of the signal recorded during the measurement is characterised by better signal-to-noise ratio (SNR).

The described nonlinear rheological properties demonstrate the very complex behaviour of the tested systems, both in the case of small amounts of water absorbed in the structure and for hydrogels with extremely large amounts. The response of the tested systems is not typical for polymer gels, but it is an intermediate form between polymer and typically colloidal systems. This behaviour is connected with the absorption of water inside the hydrogel structure, which is displaced along with the polymer chains during shear flow. In addition, it should be noted that the studied systems may have two types of bonds: Covalent and hydrogen. Covalent bonds are strong and a large amount of mechanical energy is required to destroy them. However, much less energy is needed to destroy hydrogen bonds. Therefore, systems containing a small amount of water are able to store large amounts of energy in their structure, because two types of bonds are involved in the overall phenomenon. The increase in water content causes strong spontaneous deformation of the hydrogel and breaking of weaker hydrogen bonds. This is observed as a reduction in the potential for energy accumulation of the gel network because covalent bonds begin to dominate the phenomenon. As a last resort, when the amount of bound water in the structure is critically large, the spontaneous stresses in the gel network cause complete destruction of its structure under the influence of a minimum amount introduced of mechanical energy.

## 4. Conclusions

In this work, starch-based hydrogels with excellent swelling properties have been prepared by polymerisation of partially neutralised acrylic acid and carboxymethylated high-amylose starch with various substitution degrees. In contrast to the polymerisation reaction, cross-linking occurred due to the presence of a cross-linking agent. The structural changes in starch-based hydrogels were investigated via FTIR spectroscopy. It was evident that poly(acrylic acid) chains were grafted with carboxymethylated starch particles

It has been observed that hydrogel water absorption decreases with the cross-linking agent concentration (the highest at the concentration of approximately 0.01 g). Samples with less carboxymethyl substitution were characterised by greater water absorption. The water absorption of the gel increases over a period of time of about 10 min, after which it is maintained at a constant level. A larger amount of cross-linking agent results in a higher degree of hydrogel conversion. This results in an increase in the content of monomers embedded in the network, which has a positive effect on the properties of the hydrogel.

From this research, it could be concluded that the swelling capacity of hydrogels was dependent on MBA and substitution degree.

Nonlinear rheological methods have proved to be very effective in assessing the mechanical properties of hydrogels. LAOS analysis allowed the determination of the durability of the gel structure as a function of the amount of absorbed water. In addition, it should be noted that these methods showed high sensitivity, making it possible to analyse the effect of the amount of cross-linking agent on the nonlinear deformation of the entire structure. This means that it is possible to quantify the changes in the structure of the hydrogel using LAOS methods and to unambiguously translate this information to the actual conditions prevailing when using such systems.

## Figures and Tables

**Table 1:** Reaction parameters of synthesised poly(acrylic acid)/starch hydrogels.

Sample	Carboxymethylated Starch, g	DS	MBA, g	AA, g
**CMS1SK1**	8	0.2	0.005	50
**CMS1SK2**			0.01	
**CMS1SK3**			0.03	
**CMS1SK4**			0.05	
**CMS2SK1**		0.8	0.005	
**CMS2SK2**			0.01	
**CMS2SK3**			0.03	
**CMS2SK4**			0.05	

**Table 2:** Swelling parameters of hydrogels.

Sample	x *, mol%	Swelling Characteristic				
		b, 1/mol %	*W*_max_, g_w_/g	*k*_1_, min^−1^	*K*, min^−1^	*a*
**CMS1SK1**	0.00472	350 ± 10	12.5 ± 1.5	0.73 ± 0.08	0.176 ± 0.016	1.54 ± 0.01
**CMS1SK2**	0.00953					
**CMS1SK3**	0.02802					
**CMS1SK4**	0.04831					
**CMS2SK1**	0.00434	1200 ± 15	33.8 ± 2	0.84 ± 0.1	1.2 ± 0.1	0.88 ± 0.01
**CMS2SK2**	0.00878					
**CMS2SK3**	0.02765					
**CMS2SK4**	0.04717					

* x = [MBA]/[monomers].
